# Calcium-Sensing Receptor and Aquaporin 2 Interplay in Hypercalciuria-Associated Renal Concentrating Defect in Humans. An In Vivo and In Vitro Study

**DOI:** 10.1371/journal.pone.0033145

**Published:** 2012-03-05

**Authors:** Giuseppe Procino, Lisa Mastrofrancesco, Grazia Tamma, Domenica Rita Lasorsa, Marianna Ranieri, Gilda Stringini, Francesco Emma, Maria Svelto, Giovanna Valenti

**Affiliations:** 1 Department of Biosciences, Biotechnologies and Pharmacological Sciences and Center of Excellence in Comparative Genomics, University of Bari, Bari, Italy; 2 “Bambino Gesù” Pediatric Hospital, Rome, Italy; INSERM, France

## Abstract

One mechanism proposed for reducing the risk of calcium renal stones is activation of the calcium-sensing receptor (CaR) on the apical membranes of collecting duct principal cells by high luminal calcium. This would reduce the abundance of aquaporin-2 (AQP2) and in turn the rate of water reabsorption. While evidence in cells and in hypercalciuric animal models supports this hypothesis, the relevance of the interplay between the CaR and AQP2 in humans is not clear. This paper reports for the first time a detailed correlation between urinary AQP2 excretion under *acute* vasopressin action (DDAVP treatment) in hypercalciuric subjects and in parallel analyzes AQP2-CaR crosstalk in a mouse collecting duct cell line (MCD4) expressing endogenous and functional CaR. In normocalciurics, DDAVP administration resulted in a significant increase in AQP2 excretion paralleled by an increase in urinary osmolality indicating a physiological response to DDAVP. In contrast, in hypercalciurics, baseline AQP2 excretion was high and did not significantly increase after DDAVP. Moreover DDAVP treatment was accompanied by a less pronounced increase in urinary osmolality. These data indicate reduced urinary concentrating ability in response to vasopressin in hypercalciurics. Consistent with these results, biotinylation experiments in MCD4 cells revealed that membrane AQP2 expression in unstimulated cells exposed to CaR agonists was higher than in control cells and did not increase significantly in response to *short term* exposure to forskolin (FK). Interestingly, we found that CaR activation by specific agonists reduced the increase in cAMP and prevented any reduction in Rho activity in response to FK, two crucial pathways for AQP2 translocation. These data support the hypothesis that CaR–AQP2 interplay represents an internal renal defense to mitigate the effects of hypercalciuria on the risk of calcium precipitation during antidiuresis. This mechanism and possibly reduced medulla tonicity may explain the lower concentrating ability observed in hypercalciuric patients.

## Introduction

The incidence of renal calcium stones has risen steadily over the past 30 years to become the main cause of hospitalization for uro-nephrologic reasons [1]. Stone formation is associated with an increased risk of hypertension, bone disease and chronic kidney diseases [1], [2], [3].

Urinary saturation may be the most important factor in stone pathogenesis and is strictly correlated to water reabsorption in the kidney. The kidney is a key organ regulating both water and calcium homeostasis, and its ability to sense extracellular calcium levels in both the urinary filtrate and the interstitial fluid is due to the extracellular Calcium–Sensing Receptor (CaR), which is expressed in multiple sites along the nephron [4].

Specifically, CaR protein is expressed in the apical membrane of the proximal convoluted and proximal straight tubules, at the basolateral membrane of the medullary and cortical thick ascending limbs and distal convoluted tubule, in some cells of the cortical collecting duct and at the apical membrane of the inner medullary collecting duct [4], [5], [6].

The apically located CaR in the proximal tubules appears to directly attenuate parathyroid hormone (PTH)-induced inhibition of phosphate reabsorption by proximal tubules and inhibits PTH-dependent phosphate uptake. Activation of distal tubular CaR, which is located basolaterally, directly inhibits tubular calcium and magnesium reabsorption. Thus hypercalcemia, in addition to indirectly increasing renal calcium excretion as a result of lowering PTH levels, also directly promotes calciuria.

In the collecting duct, CaR is expressed in the apical membrane, thus implying that they might be activated by urinary calcium. Evidence in animal models and in cell culture strongly suggest that activation of CaR expressed in the collecting duct epithelial cells reduces the expression of the vasopressin-sensitive water channel aquaporin-2 (AQP2) and thereby the rate of water reabsorption [7], [8], [9]. The AQP2 water channel translocates from intracellular vesicles to the apical membrane in response to an acute increase in circulating vasopressin. Water exits the cells via basolateral AQP3 and AQP4 [10], [11].

Hypercalciuria is often present in stone formers, probably due to a combination of genetic predisposition and diet [12], [13], [14]. High calcium delivery to the collecting duct would be predicted to limit local AQP-mediated water reabsorption, protecting against intratubular deposits and stone formation [15], [16], [17], [18]. While evidence supporting this hypothesis have been provided in cells and in hypercalciuric animal models, the relevance of this mechanism in humans is questioned. In fact, while hypercalciuric animals exhibit extreme hypercalciuria, humans with hypercalciuria most often have urine calcium concentrations of around 6 mM, i.e. within the range of human urine pH, and so would only weakly stimulate CaR (EC_50_ for calcium of human CaR around 6 mM at pH 5.5 to 6.5). As a consequence, CaR in hypercalciuric subjects are expected to be stimulated mainly under *acute* vasopressin action when the calcium concentration rises due to water reabsorption.

A crucial point in this context is therefore to distinguish between the short-term effect (within minutes) of a rise in luminal calcium likely occurring during antidiuresis in response to acute vasopressin action on the AQP2-mediated water reabsorption, and the long-term effect on AQP2 expression in cells chronically exposed to high luminal calcium. Previous studies in both cell and animal models have tended to address the latter point.

In order to investigate whether stimulation of the collecting duct apical CaR exerts a protective effect against intratubular calcium precipitation in humans, in the present contribution we evaluate the short-term effect of vasopressin action on AQP2 trafficking in the presence of high calcium. The work was conducted in parallel in hypercalciuric patients and in a mouse collecting duct cell line stably transfected with AQP2 (MCD4) expressing endogenous and functional CaR [19]. The results obtained support the hypothesis that in hypercalciuric patients impaired AQP2 targeting to the membrane due to activation of luminal CaR signaling might explain the defect in the kidney's concentrating ability under acute vasopressin action. The resulting reduced water absorption may be a renal defense against urinary calcium supersaturation.

## Results

### Diuresis, electrolytes, urine osmolality and AQP2 excretion evaluations in hypercalciuric patients subjected to the DDAVP test

24 h urine flow rate were measured in patients (mean age 8.6±3.9 years) with a past history of hypercalciuria that was defined by a urinary Ca/creat ratio >0.2 mg/mg on 3 separate samples obtained during a 24 h period and had been investigated after presenting with hematuria or nephrolithiasis at the “Bambino Gesù” Pediatric Hospital in Rome, Italy.

Patients enrolled in the study (n = 53) were seen on a scheduled outpatient visit where a complete tubular function, including a DDAVP test was performed. Patients were then reclassified as hypercalciuric (n = 21) or normocalciuric (n = 32) based on their urinary calcium excretion measured on samples obtained during the DDAVP test. No patient had evidence of acidosis or serum electrolyte abnormalities. Patient characteristics and the initial urinary parameters at baseline, before receiving DDAVP are reported in Table 1. As shown, the two cohorts were well matched for age, gender, body mass index and presenting symptoms.

**Table 1:** Patient characteristics and baseline urinary parameters.

	Units	Hypercalciuric	Normocalciuric	p
*Patient characteristics*				
Gender	M∶F	12∶9	20∶12	ns
Age	Years	8.2±3.8	8.9±4.1	ns
Height	SDS	0.12±0.94	0.41±1.02	ns
Weight	SDS	0.27±0.85	0.71±1.55	ns
BMI	SDS	0.30±0.91	0.79±1.82	ns
Hematuria/Nephrolithiasis(*)	n	14/7	24/8	ns
Urine output	ml/m^2^/24 h	1125±351	867±400	ns
*Baseline urinary parameters*				
UOsm	mOsm/Kg	713±247	860±250	<0.05
UCreat	mg/dl	66±45	112±72	<0.01
UUN	mg/dl	777±390	1147±429	<0.001
FeNa	%	0.69±0.49	0.51±0.29	ns
FeK	%	15.0±0.12	11.3±7.3	ns
FeCl	%	1.21±0.85	0.95±0.70	ns
FeCa	%	1.96±1.09	0.52±0.52	<0.001
UCa/UCreat	mg/mg	0.36±0.15	0.08±0.06	<0.001
UMg/UCreat	mg/mg	0.17±0.09	0.13±0.07	ns
TRP	%	97.0±2.2	98.2±2.8	ns
FeUA	%	7.3±4.1	6.2±2.7	ns
UGluc/UCreat	mg/mg	0.03±0.04	0.02±0.02	ns
UProt/UCreat	mg/mg	0.23±0.13	0.20±0.16	ns

Abbreviations: M = male; F = female; SDS = standard deviation score; UOsm = urine osmolarity, UCreat = urine creatinine, UUN = urine urea nitrogen; FeNa = fractional excretion of sodium; FeK = fractional excretion of potassium; FeCl = fractional excretion of chloride; FeCa = fractional excretion of calcium; UCa/UCreat = urinary calcium:creatinine ratio; UMg/UCreat = urinary magnesium:creatinine ratio; TRP = tubular reabsorption of phosphorus; FeUA = fractional excretion of uric acid; UGluc/UCreat = urinary glucose:creatinine ratio; UProt/UCreat = urinary protein:creatinine ratio.

(*) numbers indicate the presenting symptom or finding.

Hypercalciuric patients had a significant lower baseline urine osmolarity suggesting that children with persisting hypercalciuria had a lower urine concentration ability compared to those that had normalized their urinary calcium excretion after the initial diagnosis. No significant difference was found in the 24 h urine output between the two groups although a trend for a higher urine output was observed in hypercalciuric patients.

AQP2 excretion was next monitored in response to intranasal 20 µg DDAVP administration. Urinary AQP2 was measured by ELISA as previously described [20], [21], [22] in 3 urine samples collected before and within 2 hours of DDAVP administration (urine samples were also collected at 3 and 4 hours for electrolyte measurements).

**Figure 1:**
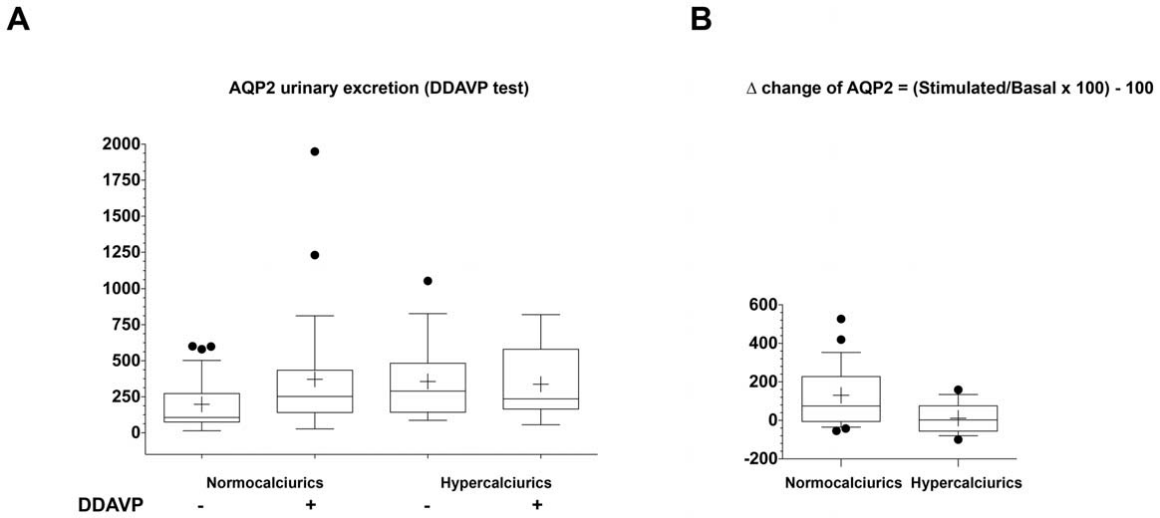
AQP2 excretion during urinary concentration test. For the urinary concentration test, urinary AQP2 excretion and osmolality were measured on samples obtained hourly after 20 µg intranasal DDAVP administration. AQP2 urinary excretion. (A) In normocalciuric children, DDAVP administration resulted in a significant increase in urinary AQP2 excretion. Hypercalciuric children had a high basal AQP2 excretion and DDAVP administration did not result in a significant increase in urinary AQP2 excretion. Data were analyzed by Wilcoxon Signed Rank ANOVA test for paired non parametric data (*p = 0.0023). (B) Comparison of basal AQP2 excretion in hypercalciurics and normocalciurics. patients. In hypercalciurics a significant higher AQP2 excretion was observed (p = 0.036 ANOVA test).

In normocalciuric children, DDAVP administration resulted in a significant increase in AQP2 excretion, (from 198,16±35,46 fmol/mg Ucre to 371,79±78,34 fmol/mg Ucre, p<0.005). In contrast, in hypercalciuric children, baseline AQP2 excretion was high (356,32±56,23 fmol/mg Ucre) and did not significantly increase after DDAVP administration (337,29±48,95 fmol/mg Ucre, n.s.) (Fig. 1A). ANOVA test, using calciuria as the nominal independent variable and urine AQP2 (before and after DDAVP admnistration) as the continuous dependant variable indicates that calciuria excretion significantly modulates urine AQP2 levels (F-value:4.53; p = 0.036).

To distinguish between the effect of calciuria on basal AQP2 from DDAVP-stimulated response, the Bonferroni/Dunn post hoc test was used and showed a highly significant effect of urine calcium concentration on basal AQP2 levels (p = 0.0073), but no significant effect on AQP2 levels following DDAVP stimulation (Fig. 1B).

**Figure 2:**
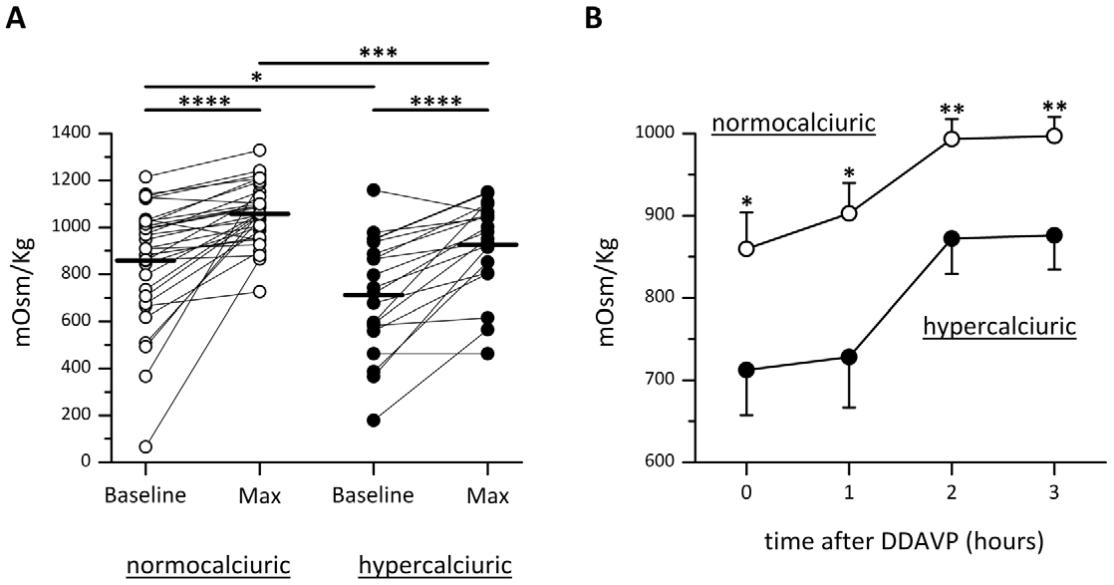
Urinary osmolality during DDAVP test. (A) Baseline and maximal urine osmolality after DDAVP test in normocalciuric and hypercalciuric patients. (B) Urinary osmolality in samples collected hourly after DDAVP administration. In hypercalciurics, DDAVP treatment was accompanied by a significantly lower increase in urinary osmolality, indicating a reduced urinary concentrating ability in response to vasopressin in hypercalciuric subjects. The values obtained were compared by one-way Anova and Tukey's multiple comparison test (*P<0.005, **P<0.001, ***P<0.0001.).

The increase in AQP2 excretion after DDAVP treatment was paralleled by an increase in urinary osmolality (mean values 1057±23 mOsm/Kg) consistent with a physiological response to DDAVP associated with AQP2 insertion into the luminal membrane of collecting duct epithelial cells (Fig. 2A and B). In hypercalciurics, DDAVP treatment was accompanied by a less pronounced increase in urinary osmolality, which remained relatively low (mean value 926±43 mOsm/Kg, Fig. 2A and B; p<0.005 vs normocalciuric). These data indicate a reduced urinary concentrating ability in response to vasopressin in hypercalciuric subjects. The reason for a high baseline AQP2 excretion in hypercalciuric subjects is unclear. Hypothetically, these observations may reflect higher cell-surface AQP2 expression at lower vasopressin levels.

The apparent discrepancy of high baseline AQP2 excretion associated with mild 24 h polyuria might be due to a reduced cortico-medullary gradient in hypercalciurics. Therefore the fractional excretion of the principal electrolytes was evaluated in hypercalciuric vs normocalciuric children at T0 and in the 3 hours following DDAVP administration, as shown in Table 2. As expected, the fractional excretion of calcium (FeCa) was significantly higher in hypercalciurics at all three tested time points (T0, T1 and T2) in accordance with calcium loss in those patients. Sodium fractional excretions (FeNa) was not statistically different between the two groups although a trend for a higher FeNa was observed in hypercalciuric patients.

**Table 2:** Fractional excretion of sodium, chloride, potassium and calcium during DDAVP testing.

	Group	Baseline	1 hour	2 hours	3 hours
FeNa (%)	Hypercalciuric	0,69±0,49	0,60±0,47	0,66±0,47	0,67±0,48
	Normocalciuric	0,50±0,29	0,47±0,24	0,51±0,51	0,52±0,34
FeK (%)	Hypercalciuric	15,0±12,2	13,6±6,5	13,7±9,0	12,7±9,4
	Normocalciuric	11,3±7,3	14,1±8,4	13,4±13,4	12,6±7,8
FeCl (%)	Hypercalciuric	1,21±0,85	1,11±0,83	1,11±0,81	1,12±0,82
	Normocalciuric	0,95±0,70	1,03±0,69	1,01±1,01	0,92±0,61
FeCa (%)	Hypercalciuric	1,73±0,78	1,84±0,87	1,69±0,72	1,51±0,47
	Normocalciuric	0,52±0,52*	0,47±0,52*	0,46±0,51*	0,40±0,51*

Abbreviations:; FeNa = fractional excretion of sodium; FeK = fractional excretion of potassium; FeCl = fractional excretion of chloride; FeCa = fractional excretion of calcium.

Data are expressed as mean ± SD;

(*) p<0.0001 Hypercalciuric vs Normocalciuric.

### CaR signaling activation and AQP2 trafficking in MCD4 cells

For a better evaluation of AQP2 constitutive as well as regulated trafficking in collecting duct renal cells under CaR activation, we analyzed the effect of CaR signaling in MCD4 cells stably expressing AQP2 [20]. We recently demonstrated that these cells endogenously express functional CaR protein [19]. In fact, in MCD4, high Ca^2+^ (5 mM), gadolinium (Gd^3+^) as well as the specific allosteric modulator of CaR, NPS-R 568 [23] caused a rapid increase in intracellular calcium, confirming that the CaR expressed is a functional receptor [19].

**Figure 3:**
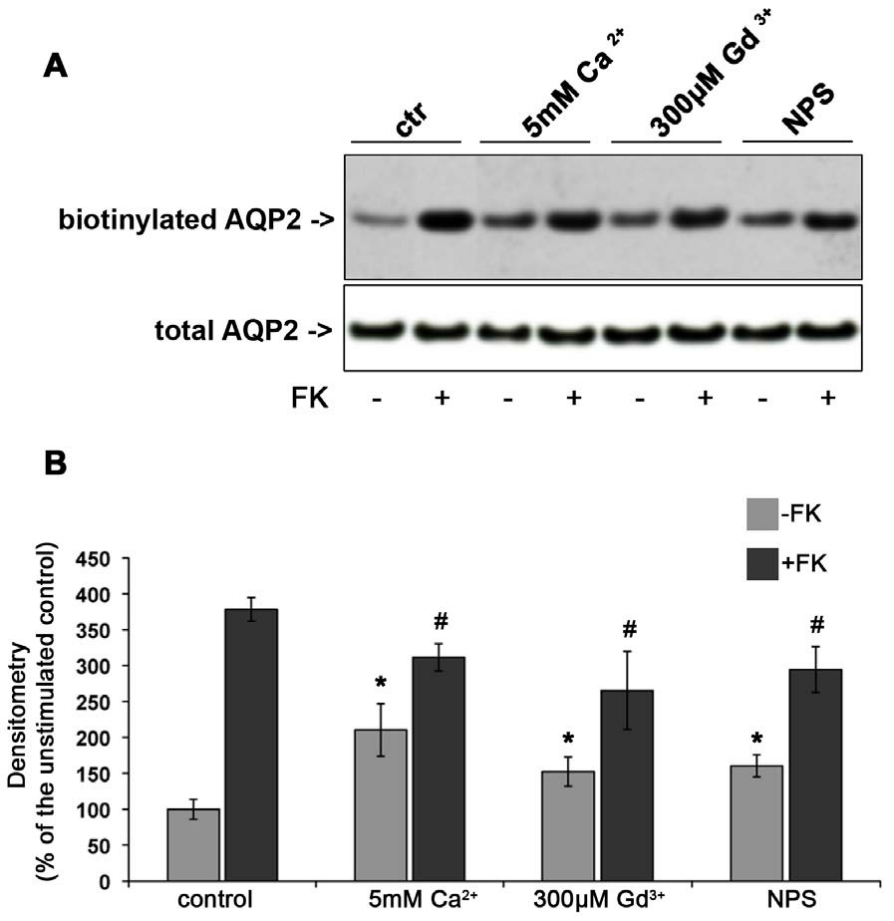
Effect of CaR signaling on AQP2 trafficking in MCD4 cells. **Apical surface biotinylation.** (A) MCD4 cells were preincubated with 5 mM Ca^2+^, 300 µM Gd^3+^ or 10 µM NPS-R 568 then exposed to FK10^−4^ M or left under control conditions. Apical membrane-expressed AQP2 was quantitated by apical surface biotinylation. FK-induced AQP2 membrane accumulation was significantly reduced in the presence of CaR agonists. CaR agonists induced a mild increase in AQP2 membrane expression even in the absence of FK stimulation. The total amount of AQP2 in the starting preparation was comparable in each experimental condition (total AQP2). (B) Densitometric analysis of the 29 kDa biotinylated AQP2 band. Results are expressed as mean values ± SEM. The values obtained in five independent experiments are expressed as percentages of the basal condition. Data were compared by one-way Anova and Tukey's multiple comparison test (* P<0.05 relative to ctr, # P<0.05 relative to FK).

To evaluate whether CaR signaling might modulate vasopressin-induced AQP2 trafficking, AQP2 accumulation at the plasma membrane was semi-quantitated by cell-surface biotinylation in response to the cAMP elevating agent forskolin (FK) under different experimental conditions. The total amount of AQP2 in the starting preparation was comparable in each experimental condition (Fig. 3A, total AQP2).

Exposure to FK caused a significant (nearly fourfold) increase in cell-surface accumulation of AQP2 (Fig. 3A and 3B). Moreover, when FK stimulation was performed in the presence of either 5 mM external calcium, or gadolinium or NPS-R 568, the FK-induced AQP2 membrane accumulation was significantly reduced with respect to unstimulated cells (Fig. 3A and 3B).

Another interesting result was the observation that, compared with untreated cells, treatment with either 5 mM calcium or with CaR agonists (gadolinium or NPS-R 568) resulted in a significant increase in the cell-surface abundance of AQP2 in the absence of FK stimulation. This finding suggests that CaR stimulation ‘per se’ causes AQP2 accumulation at the plasma membrane.

**Figure 4:**
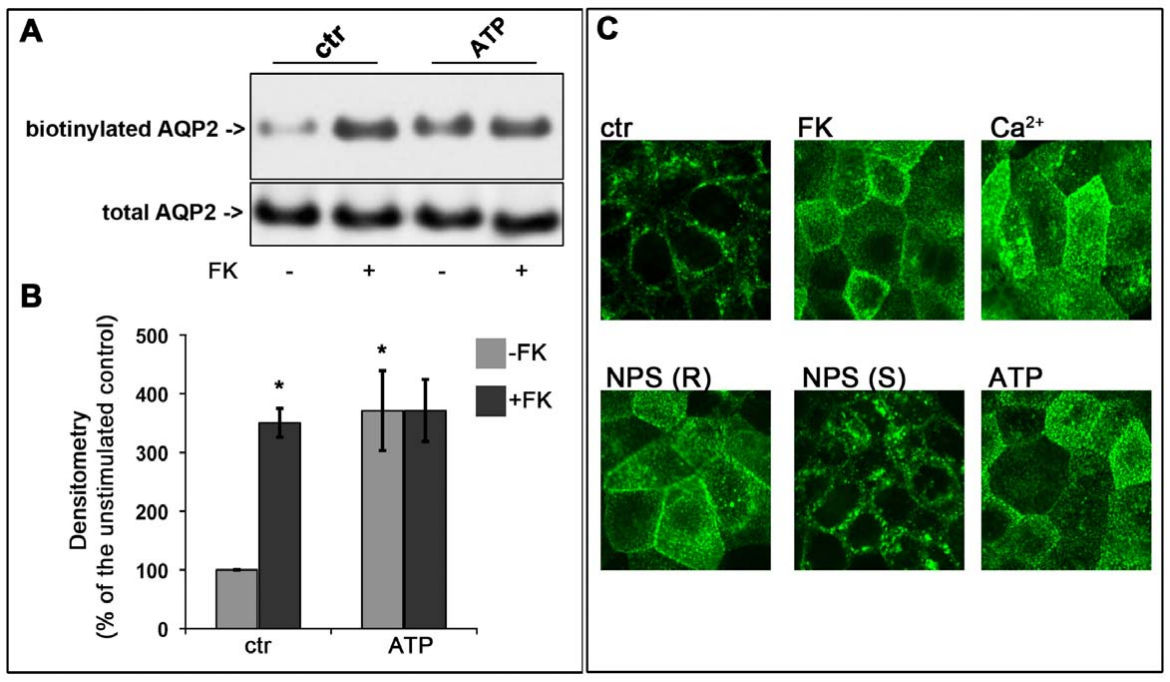
Effect of ATP stimulation on AQP2 trafficking in MCD4 cells. (A) MCD4 cells were preincubated with 100 µM ATP or used under control conditions and then stimulated with FK 10^−4^ M. The amount of apical AQP2 was quantitated by apical surface biotinylation. ATP caused AQP2 membrane accumulation comparable to that found in FK treated cells. The total amount of AQP2 in the starting preparation was comparable in each experimental condition (total AQP2). (B) Densitometric analysis of the 29 kDa biotinylated AQP2 band. Results are expressed as mean values ± S.E.M. The values obtained in three independent experiments are expressed as percentages of the basal condition. Data were compared by one-way Anova and Tukey's multiple comparison test (* P<0.05 relative to ctr.) (C) Immunolocalization of AQP2 and analysis by confocal microscopy. MCD4 cells were grown on permeable support to full confluence fixed and immunostained with antibodies against AQP2. In resting cells, AQP2 was mainly detectable in sub-apical vesicles (ctr). After FK treatment, AQP2 localized to the apical membrane (FK). A similar apical localization was observed in cells exposed to 5 mM calcium (Ca^2+^) or to NPS-R 568 (NPS R) or to ATP (ATP) treatments. By contrast, no AQP2 redistribution was observed after exposure to the inactive NPS enantiomer NPS-S (NPS-S).

In MCD4 cells, since acute exposure to each of the three agents (high calcium, gadolinium or NPS-R 568) causes an increase in intracellular calcium [19], cell-surface biotinylation experiments were performed in parallel in cells treated with a maximal dose of ATP (100 µM) known to induce increases in intracellular calcium ion levels via activation of the ATP-gated calcium channels (P2Y) [24]. The results obtained revealed that short-term exposure of cells to ATP caused a significant accumulation of AQP2 at the plasma membrane, comparable to that obtained with FK stimulation (Fig. 4 A and B). The total amount of AQP2 was comparable in each experimental condition indicating that the increase of biotinylated AQP2 was a consequence of real translocation to the plasma membrane. Immunolocalization experiments and analysis by confocal microscopy confirmed that ATP, as well as either high calcium or NPS-R 568, caused AQP2 relocation to the plasma membrane comparable to that observed with FK stimulation (Fig. 4 C). The effect was not observed using the S enantiomer of 568, which is 30- to 100-fold less potent than the R enantiomer in activating the CaR (Fig. 4 C) [25].

This data supports the hypothesis that intracellular calcium increase associated with CaR activation promotes AQP2 vesicle fusion to the plasma membrane. Moreover, FK stimulation did not result in a significant additional increase in AQP2 abundance at the plasma membrane.

### Extracellular Ca2+ or CaR Agonists reduced forskolin-induced accumulation of cAMP

Exposure to the cAMP-generating agent FK increased cyclic nucleotide accumulation (cAMP) by about threefold in MCD4 cells. Interestingly, in MCD4 cells, when FK stimulation was performed in association with CaR agonists (high external calcium or gadolinium or NPS-R 568), a strong reduction in cAMP accumulation was observed (Table 3). Taken together, these data indicate that CaR-signaling counteracts the effect of FK on cAMP synthesis, resulting in reduced cAMP levels.

**Table 3:** Measurements of intracellular cAMP levels.

*Conditions*	*n = 3*
Control	100%
forskolin	322±8%^a^
Ca^2+^(5 mM)	115±17%
Ca^2+^(5 mM)+forskolin	223±25%^b^
Gd^3+^(300 µM)	114±6%
Gd^3+^(300 µM)+forskolin	213±15%^b^
NPS R(10 µM)	125±6%
NPS R(10 µM)+forskolin	198±11%^b^

MCD4 cells were treated with 5 mM Ca^2+^, 300 µM Gd^3+^ or 10 µM NPS-R 568 for 30 minutes and then stimulated with FK 10^−4^ M for 20 min at 37°C. Results are expressed as % of the value found in the indicated relative controls.

a P<0.05 vs control.

b P<0.05 vs forskolin.

### Rho activity in cells exposed to CaR agonists

cAMP-dependent AQP2 trafficking is regulated by several proteins including small GTPases of the Rho family controlling cytoskeletal dynamics. RhoA has been shown to be a major regulator of actin assembly controlling AQP2 trafficking [26], [27]. To evaluate whether altered AQP2 trafficking observed in cells exposed to CaR agonists is associated with changes in Rho activity, this was measured by Fluorescence Resonance Energy Transfer (FRET).

**Figure 5:**
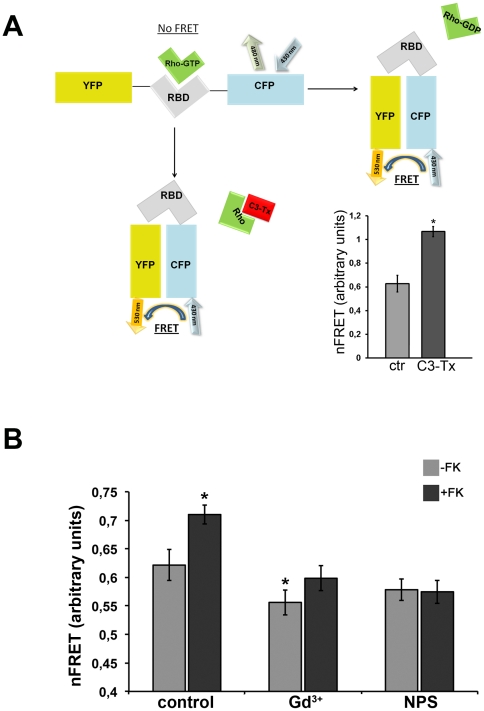
RhoA activity in cells exposed to CaR agonists. **A. Scheme of the Raichu-RBD probe mechanism.** Raichu-RBD contains YFP and CFP separated by rhotekin-RBD (RBD). Active RhoGTP binds RBD, separating the donor (CFP) from the acceptor (YFP) thus reducing FRET. As internal control MCD4 cells were incubated for 3 hours with C3 toxin (1 µg/ml), which inactivates Rho proteins, and FRET signals was recorded. As shown in the figure, C3 toxin (1 µg/ml for 3 hours) (n = 124) leaded to a ‘closed’ conformation increasing FRET compared to control cells (n = 71). **B. Rho activity during CaR activation.** RhoA activity was evaluated in MCD4 cells exposed to CaR agonists. MCD4 cells were preincubated with 5 mM Ca^2+^, 300 µM Gd^3+^ or 10 µM NPS-R 568 for 30 min and then stimulated with 10^−4^ M FK or analyzed at rest. The amount of active RhoA was evaluated by FRET using a probe consisting of a Rho-binding domain (RBD) of Rhotekin sandwiched by YFP and CFP (see Methods). In this system, any increase in RhoA activity results in a decrease in FRET efficiency. In non-treated cells, FK stimulation (n = 59) caused a significant decrease in the amount of active RhoA compared to control conditions (n = 51). In cells pretreated with 300 µM Gd^3+^, RhoA activity was significantly increased (decreased FRET signal, n = 50) compared to control untreated cells. The decrease in RhoA activity in response to FK was prevented in cells preincubated either with Gd^3+^ (n = 66) or NPS-R 568 (n = 60). Values are expressed as mean ± SEM Data were compared by one-way Anova and Tukey's multiple comparison test (*P<0.05 relative to control).

To this end, we used the Raichu-RBD probe consisting of Venus (a brighter version of yellow fluorescent protein YFP) and cyan fluorescent protein (CFP) moieties separated by rhotekin-RBD (Fig. 5A). Activation of Rho therefore leads to binding to rhotekin-RBD, increased separation of the fluorophores and consequent loss of FRET (Fig. 5A). Activation of Rho led to a loss in FRET, seen as an increase in the CFP/YFP emission ratio (FRET ratio). In contrast, Rho inactivation promoted the transfer of fluorescence from the donor (CFP) to the acceptor (YFP) thus increasing the signal of FRET.

As internal control, cells were transfected with this probe and treated with C3 toxin which inactivates Rho proteins *via* ADP-ribosylation. As shown in Fig. 5A (on the right), C3 toxin significantly increased FRET signal consistent with loss of Rho activity, confirming that this probe is suitable for mechanistic investigations. FRET experiment results are summarized in Fig. 5B. Compared to control cells, FK caused a significant decrease (depicted by an increase in FRET signal) in the amount of active RhoA, confirming previous findings in renal collecting duct cells under FK stimulation [27]. Activation of CaR with gadolinium but not with NPS-R increased basal RhoA activity with respect to control untreated cells. This finding is in agreement with our previous observation in MCD4 cells, demonstrating that gadolinium (and not NPS) induced intracellular calcium oscillation associated with activation of the Rho-ROCK pathway [19]. Of note, exposure to either gadolinium or NPS-R 568 prevented the decrease in RhoA activity in response to FK (Fig. 5B). The data obtained indicate that activation of CaR signaling in parallel with FK stimulation alters the overall equilibrium between Rho activation and Rho inactivation promoted by the cAMP elevating agent FK [27]. This may contribute to a reduced FK-dependent AQP2 accumulation on the plasma membrane.

**Figure 6:**
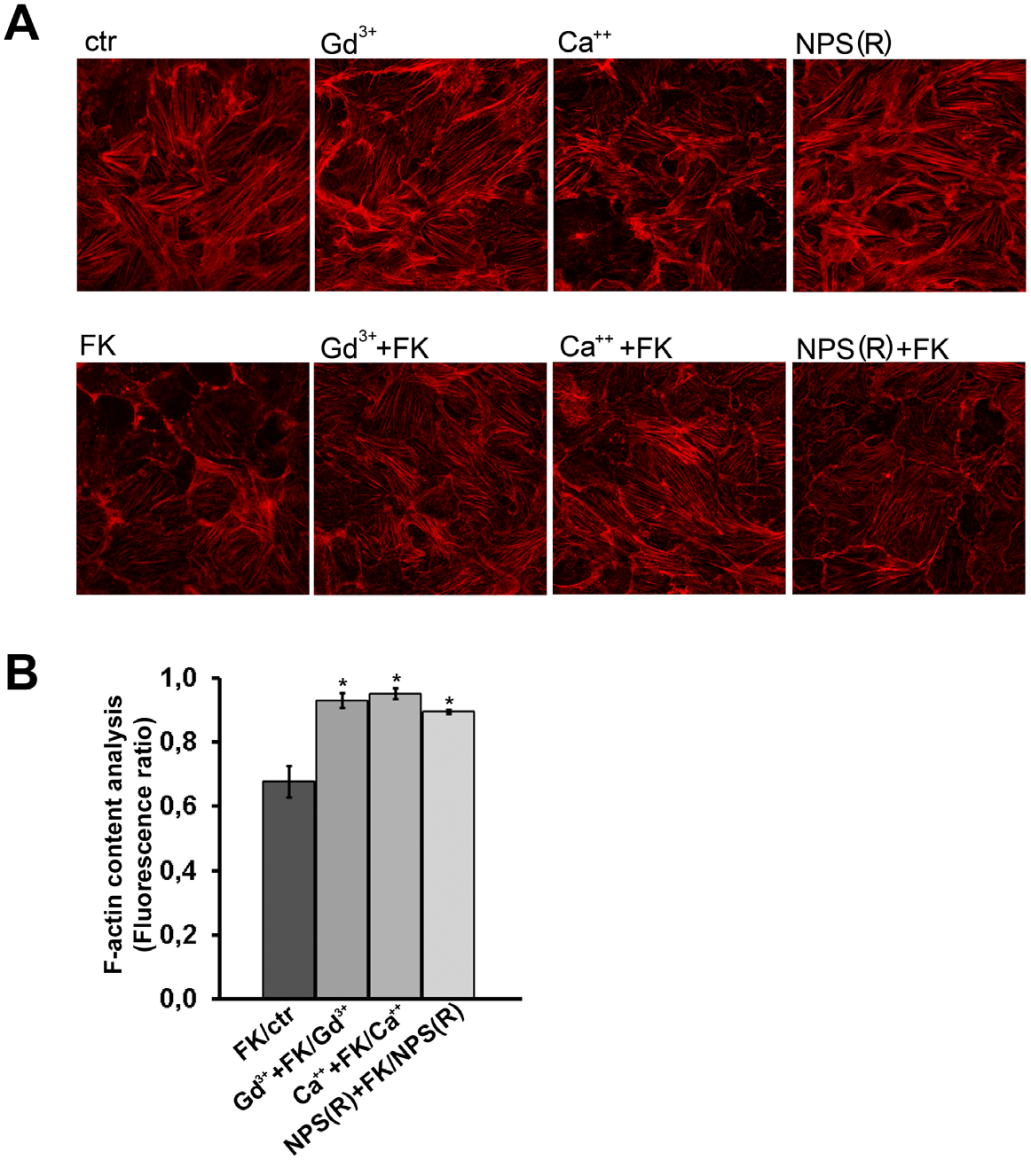
Actin cytoskeleton in cells exposed to CaR agonists. A. Visualization of actin cytoskeleton. MCD4 cells were preincubated with 5 mM Ca^2+^, 300 µM Gd^3+^ or 10 µM NPS-R 568 for 30 min and then stimulated with 10-4 M FK or analyzed at rest. Cells were fixed in PFA and stained with phalloidin Alexa Fluor-555 to visualize F-actin. Confocal pictures were taken for each experimental condition. Experiments were performed three times with similar results. B. F-actin quantization by actin polymerization assay. Confluent cells were either left untreated (CTR) or stimulated with forskolin (FK) or treated as described above with CaR agonists. After staining with TRITC-phalloidin, cells were extracted with cold methanol and the fluorescence absorbance of extracts was read (540/565 nm). The values obtained were compared by a one-way Anova and Newman-Keuls multiple comparison test (#*P*<0.05).

Since it has been shown that RhoA inhibition induces partial actin depolymerization which facilitates AQP2 accumulation at the apical plasma membrane of renal cells, the effect of CaR activation on the polymerization status of the actin cytoskeleton was next evaluated in MCD4 cells. Cells were stimulated with forskolin in the presence or absence of CaR agonists. The effect of each treatment on actin cytoskeleton was visualized by incubation with phalloidin-conjugated Alexa Fluor™-555 (Fig. 6A).

FK stimulation was associated with a partial depolymerization of the actin cytoskeleton, confirming previous findings obtained in other renal cell lines [26], [27]. Consistent with FRET data, a strong stabilization of actin cytoskeleton was observed in cells preincubated with Gd^3+^ displaying apparently a higher number of stress fibers compared to control. Stimulation with FK of both Gd^3+^ or NPS-R 568 treated cells appeared to attenuate FK-associated actin depolymerization (Fig. 6A). Semi-quantitative analysis of the amount of F-actin evaluated with the actin polymerization assay indicated that forskolin effect on F-actin content is significantly attenuated when CaR is activated with Gd^3+^, calcium and NPS-(R) (Fig. 6B).

These data, together with cAMP measurements, indicate that FK-induced AQP2 relocation to the plasma membrane is attenuated in the presence of CaR agonists.

## Discussion

The findings reported in this study indicate that high concentrations of luminal calcium attenuate short-term vasopressin-induced AQP2 trafficking via activation of the CaR in inner medullary kidney collecting duct. The data supporting this conclusion arise both from experiments performed in vitro using a well-characterized cortical principal collecting duct cell model (MCD4 cells) and from hypercalciuric patients. Specifically, this report shows the effect of CaR activation on AQP2 trafficking *within minutes*, thus not involving the already described effects of high external calcium on AQP2 expression. In fact, in a previous work, Bustamante and coworkers [7], using a mouse cortical collecting duct cell line (mpkCCDcl4), showed that long-term exposure to increasing concentrations of extracellular calcium, or treatment with CaR agonists, reduced the accumulation of both AQP2 mRNA and protein while CaR gene silencing prevented this effect. The effect on AQP2 expression in mpkCCD cells is in agreement with the observed decrease in AQP2 protein abundance in hypercalciuric rats [8], [9] supporting a direct effect of luminal calcium on AQP2 expression in collecting duct principal cells.

In this work, however, we focused our attention on the short-term effects of vasopressin on AQP2 trafficking in the presence of high external calcium. We provide *in vivo* evidence that, in the hypercalciuric patients evaluated in this study, baseline AQP2 excretion is significantly higher than in normocalciurics. In these patients, AQP2 excretion did not increase significantly after acute DDAVP administration, compared to normocalciurics. These findings, together with the observation that the 24 h urinary output, as well as the FeNa during the DDAVP test tended to be higher in hypercalciuric children, suggest the presence of a moderate urinary concentrating defect in these subjects. Consistent with these results, cell-surface biotinylation experiments in MCD4 cells revealed that membrane AQP2 expression in unstimulated cells exposed to CaR agonists was higher than in control cells and did not increase significantly in response to short-term exposure to forskolin (FK). Taken together, these data support the hypothesis that CaR–AQP2 interplay represents an internal renal defense to mitigate the effect of hypercalciuria on the risk of calcium precipitation during antidiuresis.

Calcium stones account for up to 80% of kidney stones and hypercalciuria is the most common metabolic abnormality found in calcium stone formers [29], [30], [31]. In TRPV5^−/−^ mice, Renkema and coworkers demonstrated that the risk of supersaturation produced by hypercalciuria is attenuated by activation of apical CaR, resulting in reduced AQP2 expression and higher net acid secretion though H^+^-ATPase [32]. The resulting polyuria and urine acidification protects against intratubular calcium precipitation.

Although the suggested mechanism linking CaR activation and AQP2 expression/trafficking is well supported by findings obtained in cell models and intact animals [5], [7], [8], [17], [33] whether those data may have pathophysiological relevance in humans is not clear.

Clinical evidence for an effect of luminal calcium on AQP2-mediated water reabsorption was provided for the first time in our previous studies in enuretic children [34], [35]. Hypercalciuric enuretic children treated with DDAVP and low calcium diet reduced hypercalciuria and this resulted in a reduced overnight urine output and increased nighttime AQP2 excretion and osmolality [35].

More recent studies, however, have questioned the relevance of the CaR-AQP2 interplay in humans. The first major criticism is that there is no good data supporting the idea that patients with idiopathic hypercalciuria have polyuria [36]. Secondly, luminal calcium concentrations found in humans with hypercalciuria are predicted to have only a weak stimulating effect on CaR (EC_50_ for calcium of the human CaR is around 6 mM at pH values in the range of human urine, 5.5 to 6.5) while hypercalciuric animals exhibit extreme hypercalciuria.

Regarding the first point, the hypercalciuric patients enrolled in the present contribution are in pediatric age; the prevalence of children presenting with nephrolithiasis was similar in the two groups of patients. As far as the second criticism is concerned, CaR in hypercalciuric subjects is expected to be stimulated mainly under acute vasopressin action when the calcium concentration rises due to water reabsorption. The present contribution indeed analyzes CaR-AQP2 interplay during acute vasopressin action.

Experiments described in the literature [5] on perfused isolated inner medullary collecting duct segments from rats exposed in the short term to vasopressin and either 1 or 5 mM calcium in the luminal bath, demonstrated that higher luminal calcium concentrations caused a modest but significant reduction in water permeability. Indeed, the decline in water permeability was quantitatively small (about 33%). However, this small amount might significantly reduce the risk of calcium stone formation.

In fact, in hypercalciuric subjects, a small increase in urine volume would reduce the risk of solute precipitation. Specifically, if the urine flow rate doubled during antidiuresis, the calcium oxalate ion-products would decrease by 4-fold which might be crucial in reducing the risk of calcium precipitation, as most calcium stones consist of more than 90% calcium oxalate [37]. This risk appears to be higher during the night, when plasma vasopressin is higher and the balance between calcium and water excretion is most distorted in relation to stone risk [38].

In this work, we found that in hypercalciuric children the reduced kidney concentrating ability in response to vasopressin can be at least in part explained by the reduced increase in AQP2 trafficking. However, they had significantly higher baseline AQP2 excretion than normocalciurics. The reason for this is unclear: it might reflect a higher expression of AQP2 in collecting duct epithelial cells, possibly due to high luminal calcium that in turn increases intracellular calcium and calcineurin activation in particular. Calcineurin, a calcium-dependent protein phosphatase, activates NFATc (Nuclear Factor of Activated T cell) pathways that have been shown to enhance AQP2 transcription [39]. Alternatively vasopressin levels in hypercalciuric children might be higher compared to normocalciuric children thus causing an increase in AQP2 expression. Both possibilities need to be further investigated.

To explain the apparent discrepancy between higher AQP2 excretion and lower osmolarity in the baseline samples collected in hypercalciuric patients, an important point to be considered is that these children tended to have higher excretion of Na in their urines. This suggests that they may have a moderate defect in the generation of the cortico-medullary gradient, which is critical for the production of concentrated urine. Normalization of urine calcium excretion in the “normocalciuric” cohort, which included children previously diagnosed with hypercalciuria, indicates that these tubular characteristics are not necessarily constitutive, and may represent in some cases functional adaptations, for example to diet changes.

The renal cortico-medullary osmotic gradient is generated by sodium reabsorption in the thick ascending limb. Vasopressin promotes AQP2 insertion in the apical membrane of principal cells, allowing water to passively flow along this osmotic gradient (from the tubule lumen to the interstitium). Therefore, both cell-surface AQP2 abundance and hypertonic medulla are crucial to maintenance of body water homeostasis.

Of note, all the clinical data in patients found consistent support *in vitro*, in the collecting duct cell line MCD4. We show that in MCD4 cells expressing endogenous functional CaR [19] cell-surface AQP2 expression in unstimulated cells exposed to CaR agonists was higher than in control cells and did not increase significantly in response to FK. This parallels the observation in humans.

Moreover, we found that CaR activation reduced the rise in cAMP in response to FK and prevented the decrease in RhoA activity in response to FK: both events are predicted to impair AQP2 relocation to the plasma membrane.

We underline that this is the first report analyzing short-term AQP2 trafficking in collecting duct principal cells expressing endogenous and functional CaR in the presence of selective CaR (NPS-R 568) agonists. Previous data from our lab have already demonstrated that external calcium reduced AQP2 trafficking in a rabbit cell line expressing AQP2 (CD8 cells) but a definitive demonstration that this occurred through CaR signaling was lacking [33]. Our in vitro data confirm here the complexity of the molecular machinery fine-tune controlling AQP2 trafficking in the presence of the extracellular ‘third messenger’ ionized calcium which appears to serve as a negative regulator of acute vasopressin action in the collecting duct.

In conclusion, on the basis of the data from hypercalciuric patients and from MCD4 cells, we provide evidence that, during *acute* vasopressin action, luminal CaR activation in the collecting duct attenuates AQP2 mediated water reabsorption and urinary concentration in humans, which may reduce the risk of calcium salt precipitation and nephrolithiasis.

## Materials and Methods

### Patients and urinary concentration test

Children with a history of hematuria or nephrolithiasis secondary to hypercalciuria were enrolled in the study at the Bambino Gesù Hospital in Rome. Hypercalciuria was defined by a urinary Ca/Creat ratio >0.2 mg/mg on 3 separate samples obtained during the same day. All patients were tested in an outpatient clinic; a 24 h urine collection was collected prior to the outpatient visit to evaluate urinary volume. All patients underwent a 1–deamino–arginine vasopressin (DDAVP) test as part of their tubular function evaluation. Subjects were thereafter classified as “hypercalciuric” (n = 21) or “normocalciuric” (n = 32), based on the mean Ca/creat excretion measured on the collected urine samples. Therefore, also patients labeled as “normocalciuric” had a history of hypercalciuria; control patients without a history of hypercalciuria were not enrolled for ethical reasons Patients with nephrocalcinosis were excluded, as well as subjects younger than 5 years of age, because intranasal DDAVP administration and urine sample collections may be unreliable in these children. Patients were asked to record their dietary intake and received advice from a professional dietician in order to achieve similar normal salt intakes (2 mEq/Kg of body weight) and to drink approximately 1000 ml/m^2^/day. For the urinary concentration test, urinary AQP2 excretion was measured on samples obtained hourly after 20 µg intranasal administration of DDAVP.

Patient weight and fluid intake was monitored during the test and serum electrolytes were checked before initiating the test. All patients had serum electrolytes within the normal range.

Written informed consent from a parent or legal guardian was obtained prior to DDAVP testing in all patients. The study was conducted according to the principles expressed in the Declaration of Helsinki and was approved by the Ethical Committee of the Bambino Gesù Children's Hospital (Approval date: November 3, 2004; President of the ethical committee: Prof. Pierpaolo Mastroiacovo; copy of the approval letter, as well as copies of informed consent forms are available upon request).

Urine electrolytes and osmolality were measured by the hospital clinical laboratory with standard, quality-assessed techniques.

### Antibodies and reagents

Affinity-purified Anti-rabbit antibodies against C-terminus of CaR were from Chemicon (www.chemicon.com). Affinity-purified rabbit polyclonal antibodies against human AQP2 were prepared as described elsewhere [40].

All chemicals were purchased from Sigma (www.sigmaaldrich.com). Fura-2-AM was obtained from Molecular Probes (www.invitrogen.com). NPS-R 568 was a gift from Amgen. Experiments were performed with a Ringer solution containing (in mM) 140 NaCl, 5 KCl, 1 MgCl2, 1 CaCl2, 5 glucose, 10 N-2-hydroxyethylpiperazine-N′-2-ethanesulfonic acid (HEPES) (adjusted to pH 7.4 with NaOH). In experiments with high Ca2+, the ion concentration was raised with CaCl2.

### u-AQP2 measurements by ELISA (Enzyme –linked –immunosorbent –assay)

For determination of u-AQP2 excretion, urine samples were spun at 3,000 rpm for 10 min at 4°C to remove cellular debris in the presence of the following protease inhibitors: 2 mM phenylmethylsulfonyl fluoride, 1 µg/ml leupeptin, 1 µg/ml pepstatin. u-AQP2 excretion was measured as previously described by enzyme-linked immunosorbent assay [41] with some modifications. Briefly, 10 µl of urine sample were diluted to 50 µl in PBS containing 0.01% SDS, placed in a MaxiSorp 96-well microplate (www.nuncbrand.com) and incubated for 16 hrs at 4°C. In parallel wells, increasing concentrations (50, 100, 200, 300, 400, 500 and 1,000 pg/50 µl) of a synthetic peptide reproducing the last 15 amino acids of the C-terminal region of the human AQP2 were incubated as internal standard. Each sample was analyzed in triplicate. Wells were washed with washing buffer (PBS-0.1% Tween20) and incubated with a blocking solution of PBS containing 3% BSA at room temperature for 1 hr. Ten µg of affinity-purified anti-AQP2 antibodies were diluted in blocking solution (final antibody dilution 1∶1,000) and 50 µl of the solution was added to each well and incubated for 3 hrs at 37°C. Wells were then washed with washing buffer and anti-rabbit IgG conjugated with horseradish peroxidase (www.sigmaaldrich.com) was added to each well and incubated for 1 hr at 37°C.

After five washings with washing buffer, 50 µl of the substrate solution [2,2′-azino-bis(3- ethylbenzthiazoline-6-sulfonic acid); www.sigmaaldrich.com] were added to each well and incubated for 30 minutes in the dark. Absorbance was measured with a microplate reader (Model 550, www.bio-rad.com) at 405 nm. u-AQP2 excretion was expressed as fmol/mg CreU.

### Cell culture

Mouse cortical collecting duct MCD4 cells, stably expressing human AQP2, were generated as described elsewhere [20] and maintained in DMEM/F12 1∶1 supplemented with 5% fetal bovine serum, 2 mM L-glutamine, 100 i.u./ml penicillin, 100 µg/ml streptomycin and 5 µM dexamethasone until sub-confluent. For all experiments, cells were treated with indomethacin 5×10^−5^ M O/N in the culture medium to reduce the basal cAMP concentration.

### Apical surface biotinylation

Cell monolayers were treated with or without CaR agonists for 30′ in the presence or in the absence of 10^−4^ M FK for 10 min in the culture medium at 37°C.

Filters were then rapidly washed twice in ice-cold EBS buffer for biotinylation (10 mM triethanolamine, pH9.0, 150 mM NaCl, 1 mM MgCl2, 0.1 mM CaCl2) and the apical side was incubated with 650 µl of 2.5 mg/ml EZ-link Sulfo NHS-biotin (www.piercenet.com) in EBS buffer on ice for 30 min. Filters were washed twice in ice-cold PBS-CM and unbound biotin was quenched for 10 min in quenching buffer (50 mM NH4Cl in PBS-CM) on ice. Cells were scraped from filters in 500 µl of lysis buffer (20 mM TRIS-HCl, pH8.0, 150 mM NaCl, 5 mM EDTA, 1% Triton X-100, 0.2% BSA, 1 mM PMSF, protease inhibitor cocktail), and lysates were sonicated twice for 15 sec and incubated at 37°C for 20 min. Insoluble material was pelleted at 13,000×g for 10 min and biotinylated proteins in the supernatants were precipitated for 16 h with 50 µl of Immunopure Immobilized Streptavidin beads suspension (www.piercenet.com) under rotation at 4°C. Beads from each condition were washed three times in complete lysis buffer and three times in lysis buffer without BSA. Biotinylated proteins were extracted in 30 µl of NuPAGE LDS Sample buffer (www.invitrogen.com) with 100 mM DTT, heated at 95°C for 10 min and resolved on 4–12% NuPAGE gels (www.invitrogen.com).

### Gel electrophoresis and Immunoblotting

Cellular proteins (homogenates or subcellular fractions) were separated on 4–12% NuPAGE Bis-Tris gels under reducing conditions. Protein bands were electrophoretically transferred to ImmobilonP membranes (www.millipore.com) for Western blot analysis, blocked in TBS-Tween containing 3% BSA and incubated with primary antibodies. Immunoreactive bands were detected with secondary antibody conjugated to horseradish peroxidase (HRP). After each step, the membranes were washed with TBS-Tween. Membranes were developed with SuperSignal West Pico Chemiluminescent Substrate (www.piercenet.com) and were exposed to autoradiographic Kodak Biomax XAR film, (www.sigmaaldrich.com).

Band intensities were quantitated by densitometric analysis using National Institutes of Health ImageJ (NIH) software.

### Immunofluorescence

MCD4 cells were grown on porous filters and used two days after full confluence. MCD4 cells were preincubated with 5 mM Ca^2+^, or 300 µM Gd^3+^ or 10 µM NPS-R 568 or with 100 µM ATP. Untreated cells were exposed to FK 10^−4^ M or left under control conditions. Cell monolayers were then fixed with 4% PFA in PBS for 20 min at RT and washed twice for 5 min in PBS. Cells were permeabilized with 0.1% TX-100 in PBS for 15 min at RT followed by additional washes in PBS. Antigen retrieval procedure was carried out to better expose protein epitopes. Briefly, monolayers were treated with 0.5% SDS in PBS for 5 min then washed several times in PBS. Cells were blocked with 1% BSA in PBS, then the primary antibody (anti- AQP2 affinity purified) was diluted in PBS-BSA and incubated for 2 h at RT. Bound antibody was detected with Alexa Fluor 488 conjugated donkey anti-rabbit IgG antibodies (www.invitrogen.com). All incubations were performed from both sides of the filters. Filters were excised from the support, mounted on microscope slides and viewed with a Leica TCS-SP2 confocal microscope (www.leica-microsystems.com).

### cAMP measurements

Intracellular cAMP accumulation was measured in MCD4 cells cultured in 96-well plates to confluence (10^6^ cells/well) using a cAMP enzyme immunoassay kit according to the manufacturer's protocol (Amersham cAMP Biotrak Enzymeimmunoassay (EIA) System, www.gelifescience.com).

Cells were left under control conditions or treated with 5 mM Ca^2+^, or 300 µM Gd^3+^, or 10 µM NPS-R 568 for 30′. Cells were then stimulated with 10^−4^ M FK for 20 minutes at 37°C or left under control conditions. The reaction was stopped by cooling the 96-well plates in ice. Cells were then lysed using lysis buffer provided in the kit. cAMP levels were reported as %O.D/µg proteins vs control cells.

### Rho Activity Measurement by Fluorescence Resonance Energy Transfer (FRET)

RhoA activity was measured by FRET as described [40], [42]. Briefly, MCD4 cells were transiently transfected with the Raichu-RBD construct previously described by Yoshizaki et al. [28] (kindly provided Prof. Matsuda, Osaka University, Japan). ECFP and EYFP were excited at 430 or 510 nm, respectively; fluorescence emitted from ECFP and EYFP was measured at 480/30 and 545/35 nm, respectively. FRET from ECFP to EYFP was determined by excitation of ECFP and measurement of fluorescence emitted from EYFP. Corrected nFRET values were determined according to Ritter [43].

### Actin polymerization assay

Cells were left untreated or stimulated with forskolin (10–4 M). Alternatively, cells were either preincubated with CaR agonists, as previously described in the absence or in the presence of forskolin. The treatments were stopped by adding 450 µl of 3.7% paraformaldehyde, 0.1% Triton X-100, 0.25 µM TRITC-phalloidin in 20 mM potassium phosphate, 10 mM PIPES, 5 mM EGTA and 2 mM MgCl2, pH 6.8. After staining for 1 hour the cells were washed three times with PBS and 800 µl of methanol were added overnight. The fluorescence (540/565 nm) was read in a RF-5301PC fluorimeter. The values obtained were normalized for the total amount of proteins.

#### Data Analysis

The results of the quantitative variables were expressed as mean ± standard error (SE) of the mean, unless otherwise indicated. Statistical analysis were performed by one-way ANOVA. Bonferroni/Dunn post hoc test was used to evaluate the effect of calciuria on AQP2 excretion in the enrolled patients.

*P*-values<0.05 were considered statistically significant. Data were obtained from at least three independent experiments in each experimental condition.
